# Supplementary figures and images for: Electrochemical Sensor Based on Glassy Carbon Electrode Modified with Carbon Nanohorns (SWCNH) for Determination of Cr(VI) via Adsorptive Cathodic Stripping Voltammetry (AdCSV) in Tap Water
Source: Nanomaterials (Basel). 2024 Sep 9;14(17):1465. doi: 10.3390/nano14171465 (PMC11396986; doi:10.3390/nano14171465)

## Supplementary Information

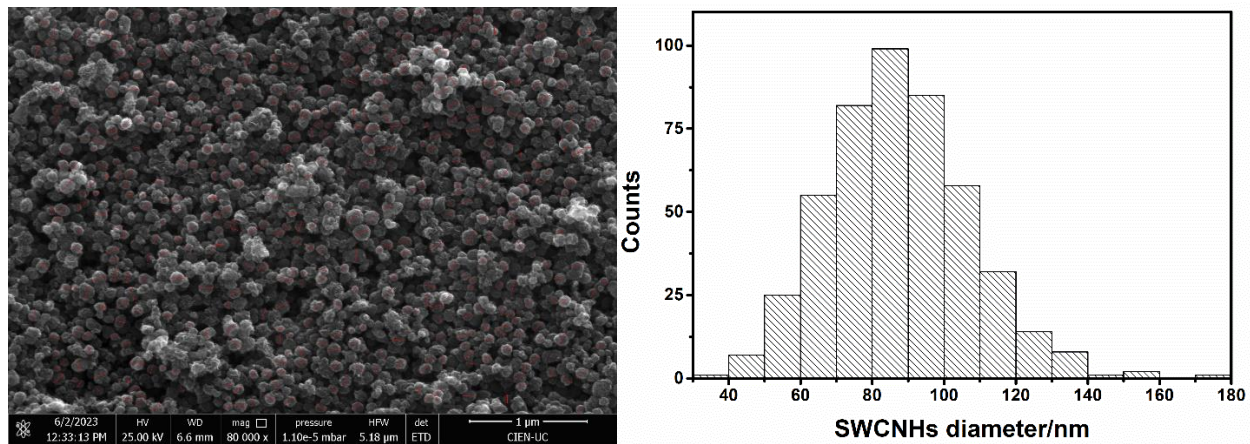

Fig. S1 Particle size histograms for SWCNHs (N=470) obtained from Fig. 1A.

Supplement: Supplementary file 1 [file nanomaterials-14-01465-s001.zip › nanomaterials-3150660-supplementary.pdf]
